# GASC1 promotes hepatocellular carcinoma progression by inhibiting the degradation of ROCK2

**DOI:** 10.1038/s41419-021-03550-w

**Published:** 2021-03-10

**Authors:** Na Shao, Jiamin Cheng, Hong Huang, Xiaoshan Gong, Yongling Lu, Muhammad Idris, Xu Peng, Belinda X. Ong, Qiongyi Zhang, Feng Xu, Chungang Liu

**Affiliations:** 1 Department of Infectious Diseases, The Second Affiliated Hospital of Chongqing Medical University, 400038 Chongqing, PR China; 2 Department of Biomedical Materials Science, School of Biomedical Engineering, Army Medical University, 400038 Chongqing, PR China; 3 Comprehensive Liver Cancer Center, The Fifth Medical Center of Chinese PLA General Hospital, 100000 Beijing, PR China; 4 Clinical Medical Research Center, Southwest Hospital, Army Medical University, 400038 Chongqing, PR China; 5 Institute of Molecular and Cell Biology, Agency for Science, Technology and Research (A*STAR), Singapore, 138673 Republic of Singapore; 6 Department of Biochemistry, Yong Loo Lin School of Medicine, National University of Singapore, Singapore, 117596 Republic of Singapore

**Keywords:** Cancer stem cells, Cancer therapeutic resistance, Gastrointestinal cancer, Oncogenes, Tumour heterogeneity

## Abstract

Hepatocellular carcinoma (HCC) is a devastating malignancy without targeted therapeutic options. Our results indicated that the histone demethylase GASC1 signature is associated with later tumor stage and poorer survival in HCC patients. GASC1 depletion led to diminished HCC proliferation and tumor growth. A distinct heterogeneity in GASC1 levels was observed among HCC cell populations, predicting their inherent high or low tumor-initiating capacity. Mechanistically, GASC1 is involved in the regulation of several components of the Rho-GTPase signaling pathway including its downstream target ROCK2. GASC1 demethylase activity ensured the transcriptional repression of FBXO42, a ROCK2 protein-ubiquitin ligase, thereby inhibiting ROCK2 degradation via K63-linked poly-ubiquitination. Treatment with the GASC1 inhibitor SD70 impaired the growth of both HCC cell lines and xenografts in mice, sensitizing them to standard-of-care chemotherapy. This work identifies GASC1 as a malignant-cell-selective target in HCC, and GASC1-specific therapeutics represent promising candidates for new treatment options to control this malignancy.

## Introduction

Hepatocellular carcinoma (HCC) is the most prevalent subtype of liver cancer and the third leading cause of cancer-related deaths^1^. The high mortality of HCC stems from the lack of suitable biomarkers for its early diagnosis and its resistance to chemotherapy. Predisposing factors for HCC development include chronically altered liver microenvironment or genetic and/or epigenetic alterations induced by cirrhosis^2,3^. The identification of novel key drivers for HCC development in the cirrhotic liver is an important unmet medical need. Chemotherapy-resistant HCC, which exhibits cancer stem cell (CSC) specific characteristics, is associated with very poor prognosis^4,5^, and often displays co-amplification and overexpression of the ROCK2 oncogene^6,7^, a key regulator of apoptosis^8^.

Amplification of genetic material is often observed in tumor cells, representing one of the underlying mechanisms leading to the activation of putative oncogenes that can contribute to tumor evolution^9^. Various types of human cancer frequently show amplification of chromosome band 9p24, which encodes lysine demethylase GASC1 (also known as KDM4C, JMJD2C, and JHDM3C), a member of the JMJD2 family^10,11^. In addition, GASC1 has been implicated in the control of cancer cell proliferation, malignant progression, and apoptosis^12,13^, especially in prostate cancer and leukemia^14,15^. Moreover, it transcriptionally activates amino acid metabolism and transport, leading to a significant increase in intracellular amino acid levels required to sustain cancer cell proliferation^16^. GASC1 knockout mice develop normally and are fertile^17^, indicating that GASC1 is not essential for physiologic tissue homeostasis and suggesting that the side effects of a therapy targeting GASC1 would be manageable. Indeed, several specific and potent GASC1 inhibitors have been developed^18^. However, to optimize the drug-target interaction, it is critical to identify the tumor types and specific molecular networks that depend on GASC1.

In this study, we tested the hypothesis that GASC1 is involved in initiating HCC cells and raising the threshold for cell death so that HCC cells become dependent on GASC1 for protection from spontaneous and chemotherapy-induced apoptosis. We identified cellular and molecular mechanisms that demonstrated the GASC1 dependency of HCC cells. Specifically, we observed that the heterogeneity of GASC1 expression determined the tumor-initiating capacity of individual cells and a functional link between GASC1 and the antiapoptotic ROCK2 pathway, as well as the effect of GASC1 on ROCK2 levels. Accordingly, SD70, a GASC1 inhibitor, significantly improved the efficacy of standard-of-care chemotherapy in both cellular and mouse xenograft HCC models.

## Materials and methods

### Cell culture

All human cell lines including Hep3B, HepG2, Huh7, PLC/PRF/5, MHCC-97H, and HEK293T were maintained in Dulbecco’s Modified Eagle’s Medium (DMEM) supplemented with 10% fetal bovine serum (FBS) (Gibco), 100 units of penicillin, and 100 mg/ml streptomycin in a sterile 37 °C incubator with a humidified 5% CO_2_ atmosphere. No mycoplasma contamination was observed in these cell lines. Cell transfection, lentiviral shRNA virus packaging and subsequent infection of various cell lines were performed according to the protocol described previously^19^. Lentiviral particles were produced in HEK293T cells and titrated onto HCC cells in medium containing puromycin (1.5 μg/ml) in order to achieve optimal knockdown of the target protein with minimal.

### Plasmids and shRNAs

HA-Ub (WT), HA-Ub (K6) only, HA-Ub (K11) only, HA-Ub (K27) only, HA-Ub (K29) only, HA-Ub (K33) only, HA-Ub (K48) only, HA-Ub (K63) only, and HA-GASC1 were purchased from Addgene. HA-Ub (K63R) was generated by cloning the corresponding cDNAs into the pCMV-HA vector via XbaI/NotI sites. pCMV-Myc-ROCK2, pCMV-Myc-ROCK2-CAT (aa 5-553), and pCMV-Myc-ROCK2-RB/PH (aa 686-1388) were generated by subcloning the corresponding cDNAs into the pCMV-Myc vector via KpnI/XhoI or XbaI/NotI sites. HA-FBXO42 and HA-FBXO42-△F-box (deleting aa 44-93) was generated by subcloning the corresponding cDNAs into the pCMV-HA vector via XhoI/BamHI sites. pCMV-Myc-ROCK2-K121R was generated using the Quick Change Q5 Site-Directed Mutagenesis Kit (NEBaseChanger) according to the manufacturer’s instructions. The pLKO.1-puro lentiviral MISSION shRNA constructs targeting endogenous GASC1 (shGASC1 CDS1 (TRCN0000022056): sense, 5′-GCCTCTGACATGCGATTTGAA-3′, and shGASC1 CDS2 (TRCN0000022055): sense, 5′-CCTTGCATACATGGAGTCTAA-3′), FBXO42 (shFBXO42 CDS (TRCN0000134822): sense, 5′-CCATCAGTGTTATCATGGTTT-3′, and a non-targeting (NT) control shRNA (TRC1/1.5) were from Sigma-Aldrich. Details of plasmid construction are available upon request.

### Drug treatments

SD70 (M60194-2s) was purchased from Xcessbio Biosciences Inc; Cycloheximide (CHX; N11534), Calpeptin (C8999), chloroquine (C6628), iodoacetamide (I1149), and N-ethylmaleimide (E-3876) were purchased from Sigma-Aldrich; Sorafenib (8705) was purchased from Cell Signaling Technology; MG-132 (S2619) was purchased from Selleck; Z-VAD-FMK (2163) was purchased from Tocris. Treatment conditions were described in detail in the text.

### Antibodies and kits

All antibodies were used at a 1:1000 dilution in 5% nonfat milk for immunoblot. PE Mouse IgG1 (555749), PE Mouse Anti-Human CD13 (560998), PE Mouse Anti-Human CD133 (566593), and PE-CF594 Mouse Anti-Human CD326 (565399) were purchased from BD Biosciences. GASC1(sc-98678), c-Myc (sc-40), and SOX9 (sc-166505) were purchased from Santa Cruz Biotechnology. Nanog (ab109250), OCT4 (ab19857), SOX2 (ab97959), FBXO42 (ab81638), and GFP (ab290) were purchased from Abcam. Cleaved caspase-3 (9664), cleaved caspase-7 (8438), cleaved caspase-9 (7237), cleaved caspase-PARP (5625), Myc tag (71D10) (2278S), HA tag (2367), ROCK1 (4035), ROCK2 (9029), BCL2 (2872), ubiquitin (3936), Ki-67(9449), p-MLC2 (3671), GAPDH (2118), Rho-GTPase Antibody Sampler Kit (9968), Active Rho Detection Kit (8820), and NF-κB p65 Antibody Sampler Kit (4767) were purchased from Cell Signaling Technology.

### CRISPR/Cas9-mediated deletion of GASC1

GASC1-KO cell lines were generated using a GASC1-specific CRISPR/Cas9/GFP (Cat# sc-403282, Santa Cruz Biotechnology) or Control CRISPR/Cas9/GFP plasmids (Cat# sc-418922, Santa Cruz Biotechnology). Hep3B cells were transfected with plasmids and selected with GFP by FACS. The selection of single cell clones was performed by serial dilution in 96-well plates, followed by immunoblot analysis of GASC1 to confirm knockout efficiency of multiple selected clones.

### High-density protein microarray screening analysis

Approximately 5 × 10^6^ of sgCtrl and sgGASC1 cells derived from the HCC cell line Hep3B were seeded into 10 cm dishes and incubated for 4 h with DMEM + 2% FBS. This was followed by a change of 5 mL serum-free DMEM and further incubation for 24 h. Cell lysates were collected for a high-density protein microarray (RayBiotech, Inc. Cat#: AAH-BLG-1000) screening analysis as per the manufacturer’s instruction.

### Immunoblot (IB) and immunoprecipitation (IP) assays

Cells were lysed with IP lysis buffer (Cat#87788, Thermo Fisher Scientific) supplemented with protease/phosphatase inhibitors (Cat#5872S, Cell Signaling Technology). Lysates were subjected to SDS-PAGE and transferred to nitrocellulose membranes. Then the membranes were incubated with various primary antibodies at 4 °C overnight, followed by incubation with HRP-conjugated anti-rabbit or anti-mouse secondary antibodies for 2 h at room temperature. Immunoreactive bands were visualized by enhanced chemiluminescence (Cat#34096, Thermo Fisher Scientific). To perform immunoprecipitation, appropriate amounts of WCL were incubated with primary antibodies (2–3 μg) overnight at 4 °C. Protein A/G sepharose beads (Cat#78610, Thermo Fisher Scientific) were then added and the incubation was continued for 3–4 h before 4× wash with IP lysis buffer. For western blot analysis, equal amounts of WCL or immunoprecipitate were resolved by SDS-PAGE and immunoblotted with indicated antibodies.

### Immunofluorescence (IF) staining

IF was performed as previously described^20^. Briefly, cells were fixed in 4% paraformaldehyde for 30 min. After three times of cold PBS wash, fixed cells were permeabilized with 0.2% Triton X-100 for 10 min, washed in PBS and then blocked in PBS supplemented with 10% goat serum. Cells were incubated with indicated primary antibody at 4 °C overnight. After three times of wash with PBS, cells were incubated with secondary antibody that was conjugated with Alexa Fluor 647 dye (Life Technologies/Molecular Probes, A32733) for 1 h at room temperature. After three times of wash with PBS, cells were mounted with vectashield mounting medium containing 40,6-diamidino-2-phenylindole (DAPI, Sigma-Aldrich, D9542) for nuclei counterstaining. Images were captured using Zeiss laser confocal microscope (LSM780).

### Immunohistochemistry (IHC) staining

Human HCC tissue collection and study approval were described previously^21^. Human HCC and adjacent matched non-tumor tissue samples were obtained from Institute of Hepatobiliary Surgery at Southwest Hospital in Chongqing, China. The use of human HCC samples and the relevant database was approved by the Army Medical University Ethics Committee and complied with all relevant ethical regulations. All tissue samples were collected in compliance with the informed consent policy. Tumor tissues were harvested, fixed in 10% formalin, and embedded in paraffin for IHC assays. IHC staining was performed using a DAKO Autostainer (DAKO, Carpinteria, CA) with DAKO LSAB+ and diaminobenzadine as the chromogen. To quantify GASC1 (sc-98678, Santa Cruz Biotechnology), Ki-67 (9441, Cell Signaling Technology) and cleaved-Caspase-3 (9664, Cell Signaling Technology) expression, we measured the immunostaining scores of GASC1, Ki-67 and cleaved-Caspase-3 as described previously^21^. Briefly, the extent of the staining, defined as the percentage of positive staining areas of tumor cells in relation to the whole tumor area, was scored on a scale of 0–4: 0; 1, 1–25%; 2, 26–50%; 3, 51–75%; and 4, 76–100%. Staining intensity was scored on a scale of 0–3: 0, negative; 1, weak; 2, moderate; and 3, strong. The overall protein expression score (range 0–12) was calculated by multiplying the positive and intensity scores.

### In vivo ubiquitination assay

HEK293T cells were co-transfected with HA-tagged ubiquitin and the respective constructs. Thirty-six hours post transfection, cells were treated with 10 μM MG-132 for 6 h. Cells were lysed in IP lysis buffer containing freshly dissolved iodoacetamide and N-ethylmaleimide (5 mM each) to inhibit deubiquitinating enzymes. Immunoprecipitation against the target protein tag was performed. Immunoprecipitants were washed five times with IP lysis buffer before being resolved by SDS-PAGE and immunoblotted with indicated antibodies.

### Flow cytometry analysis

G/EGFP+ and G/EGFP− cell populations were isolated by FACS after labeling with CD13-PE, CD133-PE, or EpCAM-PE antibodies at 4 °C for 30 min. CD13-PE, CD133-PE, or EpCAM-PE antibodies were used at the concentration recommended by the manufacturer. The stained cells were analyzed with FACS Aria II (BD Biosciences).

### Caspase-3/7 activation assays

Cells were plated in triplicate at 4000 cells/well in 96-well plates. Caspase activation was determined using the Caspase-Glo3/7 Assay (Cat#G8092, Promega), following the manufacturer’s instructions.

### Protein half-life assay

Cells were transfected or treated under indicated conditions. For protein half-life assay, cycloheximide (100 μg/ml, Sigma-Aldrich) was added to the medium. At indicated time points thereafter, cells were harvested and protein abundances were measured by immunoblot analysis.

### Mouse xenograft assays

The procedures related to animal studies were approved by the Ethics Committee of the Institutional Review Board of the Army Medical University and conformed to the NIH guidelines on the ethical use of animals. The sample size of the animals were justified by statistical considerations and statistical power analyses. The animals were randomly allocated to different experiments and outcome assessment. Hep3B (2 × 10^6^) or PLC/PRF/5 (1 × 10^6^) cells were injected subcutaneously into both flanks of 4–5-week-old male NOD/SCID mice as described previously^19,21^. At the end of the experiments, the mice were humanely killed, and each mouse’s tumor was harvested. Tumor volumes were calculated using the formula *V* = (π/6) × *a* × *b*^2^, where *a* and *b* are the tumor’s long axis and short axis, respectively.

### Therapeutic tumor model

Subcutaneous xenografts were established with the Hep3B and PLC/PRF/5 HCC cell lines. For in vivo therapeutic experiments, we followed the previously described protocol^18,22^. Briefly, when the tumor size reached between 150 and 200 mm^3^, animals were randomly distributed to groups receiving vehicle, 10 mg/kg of sorafenib via oral administration, 10 mg/kg of SD70 via i.p. injection, or both with continuous tumor monitoring until the tumor burden less than 20 mm in one dimension for 2–3 weeks. For SD70 drug preparation, SD70 powder was first dissolved in DMSO at 50 mg/mL, then diluted into 75% PEG300:25% D5W to arrive at 2.5 mg/mL.

### Human clinical data analyses

Both raw read counts and normalized read counts (sequenced with the Illumina HiSeq platform) for the The Cancer Genome Atlas (TCGA) datasets were downloaded from the Broad Institute GDAC Firehose (https://gdac.broadinstitute.org/) for HCC. Clinical information for the tumor samples were obtained using TCGAbiolinks in R^23^. Overall survival data for the liver cancer patients was obtained from the cbioportal database (https://www.cbioportal.org/). The patients were subsequently stratified into 30% top-scoring percentile (in GASC1 target gene score) versus the rest of the cohort. These stratified cohorts were used to perform Kaplan–Meier survival analyses, with the log-rank test used to determine the statistical significance. The survival analysis was performed over a 5 year (60 months) survival time frame, using the survival package (v2.44) in R.

### GASC1 target gene sets analyses

An initial set of GASC1 target genes was acquired from previous publications that examined the GASC1 target gene expression in breast cancer^24^ and esophageal cancer^17^. This initial target gene set contained 201 upregulated genes and 496 downregulated genes. To obtain a list of liver cancer specific GASC1 target genes, we performed differential gene expression analysis on TCGA HCC with matched normal samples (*n* = 50 pairs). The raw RNA-seq read counts were processed using the DESeq2 pipeline^25^ to determine the differential gene expression of the initial GASC1 target genes in the tumors relative to the paired normal samples. A filtered GASC1 target gene set (with 56 upregulated genes and 131 downregulated genes) with statistically significant differential expression (adjusted *p* value ≤ 0.05) was used to calculate the GASC1 target gene score for each TCGA HCC primary tumor (*n* = 371).

### GASC1 signature target gene score analyses

The GASC1 target gene scores were calculated based on the gene expression profiles (normalized counts) for individual primary liver tumor samples and the set of GASC1 target genes. The data was processed using ssGSEA^26,27^ from the GSVA package (v1.32)^28^ in R. This scoring was performed separately for the upregulated and downregulated GASC1 target genes. The final GASC1 target gene score was derived by subtracting the downregulated gene score from upregulated gene score. The distribution of gene scores for the liver cancers that were grouped based on clinical data were visualized with empirical cumulative distribution function (ECDF) plots with Kolmogorov–Smirnov test applied to assess statistical significance between different ECDFs. All statistical analyses were performed in R (https://www.r-project.org/).

### Quantitative real-time RT-PCR (qRT-PCR)

Total RNA was extracted using the Trizol reagent (Cat#9109, TakaRa), and the reverse transcription reaction was performed using RevertAid™ First Strand cDNA Synthesis Kits (Cat#K1622, Fermentas). Real-time PCR reaction was performed with SYBR Select Master Mix and Bio-RAD CF384 Real-Time PCR system. All procedures were performed according to the manufacturer’s instructions.

### Primer design for qRT-PCR

PrimerBank was used as the source for the primers used for PCR quantitation of mRNAs^29^, and primers used in this study are listed in Supplementary Table S1.

### Chromatin immunoprecipitation (ChIP) assay

ChIP was performed as described previously^19^. Cell lysate was sonicated and subjected to immunoprecipitation using anti-H3K36Me3 antibody or matching IgG. After extensive wash, immunoprecipitated DNA was amplified by real-time PCR. Sequence information of ChIP primers is provided in Supplementary Table S2.

### Sphere and colony-formation assays

Sphere formation assay, as well as colony-formation assay were performed as previously described^21,30^.

### Statistical analysis

Data are presented as mean ± SEM from at least three independent experiments by Student’s paired or unpaired two-tailed *t* test or two-way ANOVA, as appropriate. (*P < 0.05; **P < 0.01; ***P < 0.001, n.s., not significant). Log-rank analysis was used to determine statistical significance of Kaplan–Meier survival curves. Analyses were performed using GraphPad Prism 5.0 and SPSS 19.0 software.

## Results

### GASC1 promotes the colonization and growth of HCC

**Fig. 1:**
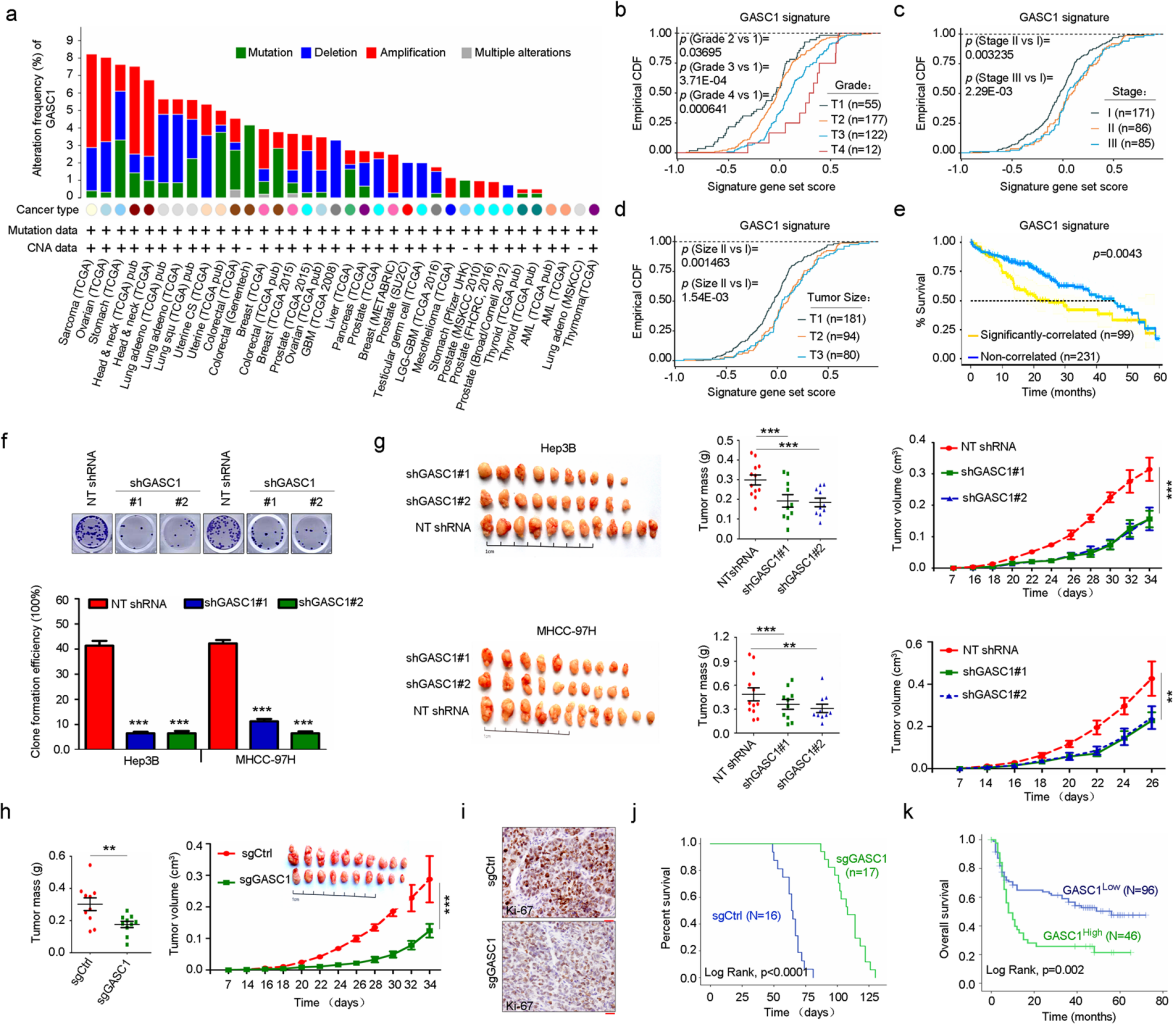
Depletion of *GASC1* suppresses HCC proliferation and tumor growth. **a** Distribution of alteration frequency in *GASC1* gene in multiple cancer types. Details of the corresponding tumor data set and the year of publication were indicated in parentheses. CNA copy-number alteration. **b** Empirical cumulative distribution function (CDF) plots showing correlation of individual tumors with GASC1 signature across various tumor grades within the HCC cohort. **c** CDF plots showing correlation of individual tumors with GASC1 signature across various clinical stages within the HCC cohort. **d** CDF plots showing correlation of individual tumors with GASC1 signature across various tumor size within the HCC cohort. **e** Kaplan–Meier survival curves comparing subjects in the TCGA HCC cohort stratified by correlation with GASC1 signature. Tumor samples were binned according to their gene expression correlation with GASC1 signature. Subjects harboring the top 30% (*n* = 99) most correlated tumors exhibited significantly decreased survival as compared to the remaining subjects (*n* = 231) from the TCGA HCC cohort. **f** Efficiency of colony-formation was examined in GASC1 shRNA (shGASC1) and Non-targeting shRNA (NT) treated Hep3B and MHCC-97H cells 2–3 weeks after plating. Data are presented as mean ± SEM of three independent experiments. ***P < 0.001 by Student’s *t* test. **g**, **h** *GASC1* depletion affects tumor growth in mouse xenograft. *GASC1* depletion was achieved by inducible shRNA treatment (**g**) or genome editing by *GASC1* CRISPR-Cas9 (**h**). Mice were sacrificed 34 days (Hep3B) or 26 days (MHCC-97H) after implantation. Tumor image (left panel) and tumor weight (middle panel) are presented. Tumor growth was measured at the indicated time points and tumors were dissected at the endpoint (*n* = 10–12 tumors) (right panel). Data are presented as mean ± SEM. **P < 0.01 and ***P < 0.001 by two-way ANOVA. **i** Representative images of Ki-67 immunohistochemistry (IHC) staining in the sgCtrl and the sgGASC1 tumor groups (H). Scale bar: 20 μM. **j** Kaplan–Meier survival curve of mice with orthotopic liver tumor induced by intrahepatic implantation of Hep3B cells transfected with shGASC1 or NT shRNA. **k** Kaplan–Meier analysis of GASC1 expression and overall survival of HCC patients (*N* = 142).

Elevated GASC1 level has been observed in multiple types of human malignancies compared to normal adjacent tissues^12–15^. In sarcoma, ovarian, colorectal, stomach, breast, lung and liver cancers, amplification of the *GASC1* locus was observed in 0.5–5% of the patients (Fig. 1a). In liver cancer patients, we found that a positive correlation between *GASC1* copy-number and mRNA levels (Supplementary Fig. 1a). An analysis of the GASC1 signature in a data set from human HCC patient samples showed a significant enrichment of the GASC1 signature in tumors of higher grade (Fig. 1b), later stage (Fig. 1c), and larger size (Fig. 1d). Ranking tumors by the strength of their correlation with the GASC1 signature allowed for stratification of all TCGA subjects with HCC into two subpopulations. The subpopulation with higher GASC1 correlation displayed significantly shorter survival times as compared to the rest of cohort (Fig. 1e). These results suggested that higher *GASC1* levels in HCC are associated with tumor progression.

Given the critical roles of GASC1 in reprogramming transcription and chromosome segregation^14,15,31^, we hypothesis that GASC1 may regulate tumor development through these mechanisms and we focused on its function in HCC progression. To evaluate the effects of GASC1 on in vitro cell proliferation and in vivo tumor growth, we first determined the endogenous levels of GASC1 in various HCC cell lines (Supplementary Fig. 1b, c) and selected a few of them as GASC1^High^ (Hep3B and MHCC-97H) and GASC1^Low^ cell lines (Huh7 and PLC/PRF/5). We found that *GASC1*-knockdown (KD) impaired the proliferation of GASC1^High^ cells but had no significant effect on GASC1^Low^ cells (Supplementary Fig. 1d). Moreover, *GASC1*-KD reduced colony formation of GASC1^High^ cell lines (Fig. 1f). Consistent with the in vitro results, silencing of *GASC1* in Hep3B and MHCC-97H cells by shRNAs significantly suppressed growth of subcutaneously implanted tumor xenograft in mice (Fig. 1g). To further validate the function of GASC1, we knocked out (KO) *GASC1* in HCC cell line Hep3B using CRISPR/Cas9 (Supplementary Fig. 1e). The isolated GASC1 KO clone showed reduced tumor growth in vivo (Fig. 1h, i). To recapitulate tumor progression in the subcutaneous xenograft model, GASC1 was over-expressed in the GASC1^Low^ cell line-PLC/PRF/5, and the overexpression of GASC1 significantly increased tumor growth (Supplementary Fig. 1f, g). Similar to the observations in the subcutaneous xenograft model, we found that in an orthotropic tumor model, the survival time was significantly longer in the GASC1 KO group, as compared with that in the control group (*P* < 0.0001, Fig. 1j). Kaplan–Meier analysis indicated that high GASC1 levels in HCC was significantly correlated with reduced overall survival (Fig. 1k). Thus, our results indicated that GASC1 plays important roles in driving HCC growth.

### The heterogeneity of GASC1 expression in HCC defines the tumor-initiating capacity of individual cells

**Fig. 2:**
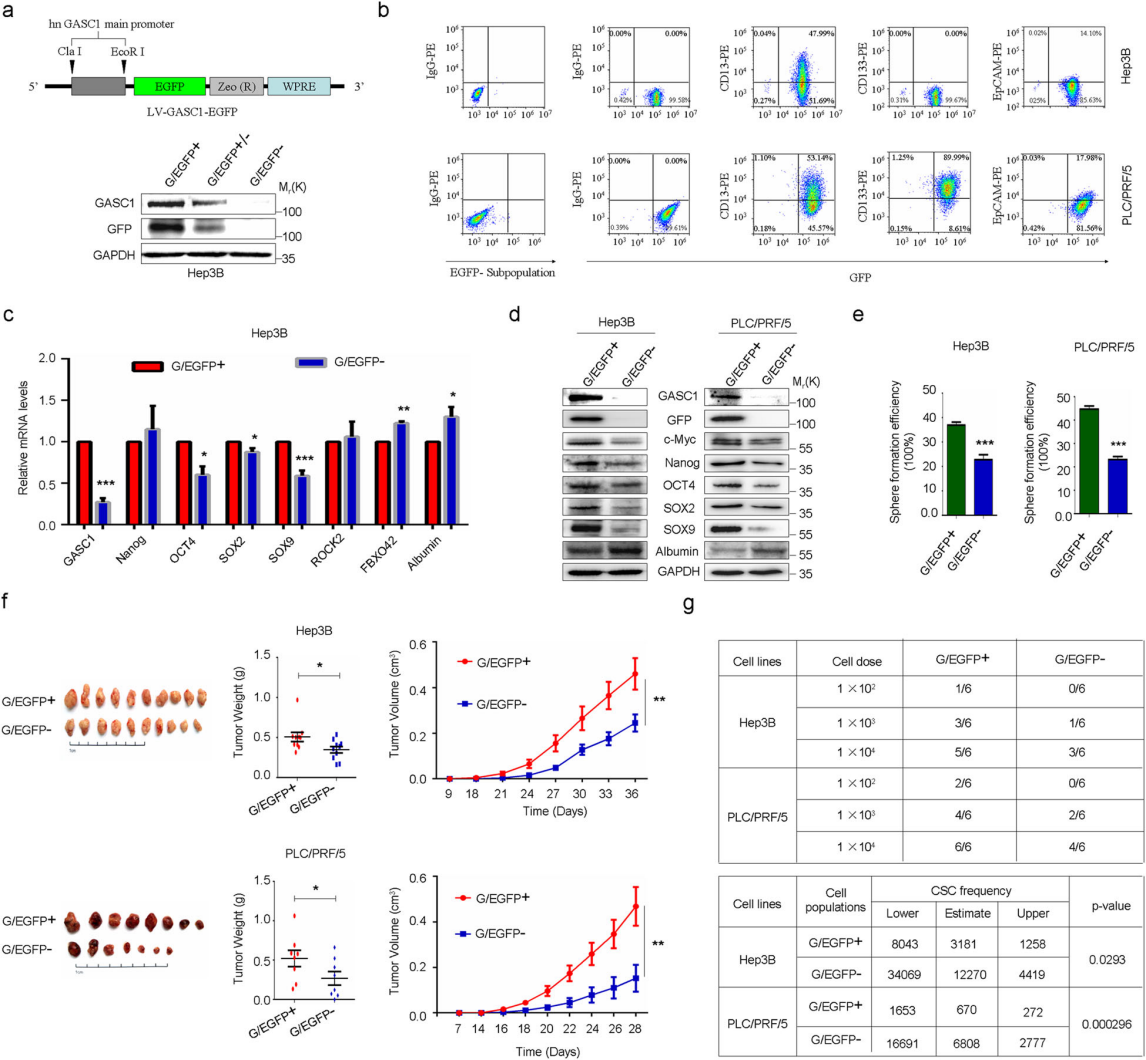
GASC1 expression defines the label-retaining CSC characterization in HCC. **a** Schematic map of lentiviral vector LV-GASC1-EGFP, in which EGFP and Zeocin expression were controlled by human GASC1 promoter (upper panel). Immunoblot (IB) of sorted populations showed a significant positive correlation between exogenous EGFP and endogenous GASC1 expression in Hep3B cells after infection with LV-GASC1-EGFP (lower panel). **b** Flow cytometric determination of the co-expression of G/EGFP+ with CD13, CD133, or EpCAM in HCC cell lines (Hep3B and PLC/PRF/5); the G/EGFP- subpopulation represents a negative control group. **c**, **d** qRT-PCR (**c**) and immunoblotting (**d**) evaluation of the mRNA and protein levels of indicated genes in FACS-sorted subpopulation (G/EGFP+ and G/EGFP−). Data are presented as mean ± SEM of three independent experiments. *P < 0.05; **P < 0.01; ***P < 0.001 by Student’s *t* test. **e** The G/EGFP+ subpopulation showed an increased oncosphere formation capacity at Day 14 of the assay. Data are presented as mean ± SEM of three independent experiments. ***P < 0.001 by Student’s *t* test. **f** Xenograft tumor growth assay of sorted G/EGFP+ and G/EGFP- subpopulation of Hep3B and PLC/PRF/5 cells in NOD/SCID mice. Tumor image (left panel) and tumor weight (middle panel) are presented. Tumor growth was measured at the indicated time points and tumors were dissected at the endpoint (*n* = 8–10 tumors) (right panel). Data are presented as mean ± SEM. **P < 0.01 by two-way ANOVA. **g** The sorted G/EGFP+ and G/EGFP− subpopulation of Hep3B and PLC/PRF/5 cells were serially diluted and injected subcutaneously in the NOD/SCID mice for extreme limiting dilution analysis (ELDA, http://bioinf.wehi.edu.au/software/elda/).

HCC tissues are highly heterogeneous and contain specific cell populations with increased tumor-initiating capacity that are referred to as CSCs or tumor-initiating cells (TICs)^32,33^. We isolated CSC and non-CSC populations using previously reported HCC CSC markers^33–36^, and found that the GASC1 levels were significantly higher in the CSCs than in the non-CSCs (Supplementary Fig. 2a). Similar observations were also made in oncosphere cells derived from HCC cell lines (Supplementary Fig. 2b). Moreover, in xenograft tumors derived from PLC/PRF/5 CSC cells prepared as we previously reported^19^, higher levels of GASC1 protein were detected as compared to those from tumors derived from non-CSC cells (Supplementary Fig. 2c). Interestingly, the depletion of GASC1 in CSCs by shRNA led to decreased liver cancer cell proliferation in vitro as examined by the CCK-8 analysis and Ki-67 IF staining (Supplementary Fig. 2d, e). Moreover, the efficiencies of clone formation and sphere formation were also reduced in liver CSCs upon GASC1 knockdown by shRNA (Supplementary Fig. 2f, g). Finally, we examined the impact of GASC1 knockdown in liver CSCs on tumor growth and initiation in vivo. In the xenograft tumor growth assay, we found that the depletion of GASC1 significantly impaired the tumor growth (Supplementary Fig. 2h). In addition, GASC1 depletion also decreased tumor initiation as examined by the limiting dilution assay (Supplementary Fig. 2i). To further examine the link between GASC1 and tumor-initiating capacity, we transformed cells using a lentiviral construct, pLV-GASC1-EGFP, which drives cytoplasmic EGFP expression under the control of the co-cloned human GASC1 promoter (abbreviated G/EGFP; Fig. 2a). FACS analysis of live G/EGFP-positive cell isolates showed that HCC cells with enhanced GASC1 expression displayed a heterogeneous CSC mass distribution (Fig. 2b). A significantly higher expression of GASC1 and a number of CSC-related genes including c-Myc, Nanog, OCT4, SOX2, and SOX9, along with a lower expression of Albumin, a marker gene for differentiation, was observed in the sorted G/EGFP-positive population, as compared to those in the G/EGFP-negative population (Fig. 2c, d). The G/EGFP-positive population showed an enhanced sphere-forming capacity in vitro (Fig. 2e) and an accelerated tumor growth in vivo (Fig. 2f). Limiting dilution analysis showed that G/EGFP-positive populations had a significantly higher proportion of CSCs, compared to that of G/EGFP-negative populations (Fig. 2g). Thus, our results indicated that HCC cell populations displayed heterogeneous levels of GASC1 and CSC enrichment.

### Posttranscriptional control of ROCK2 protein level by GASC1

**Fig. 3:**
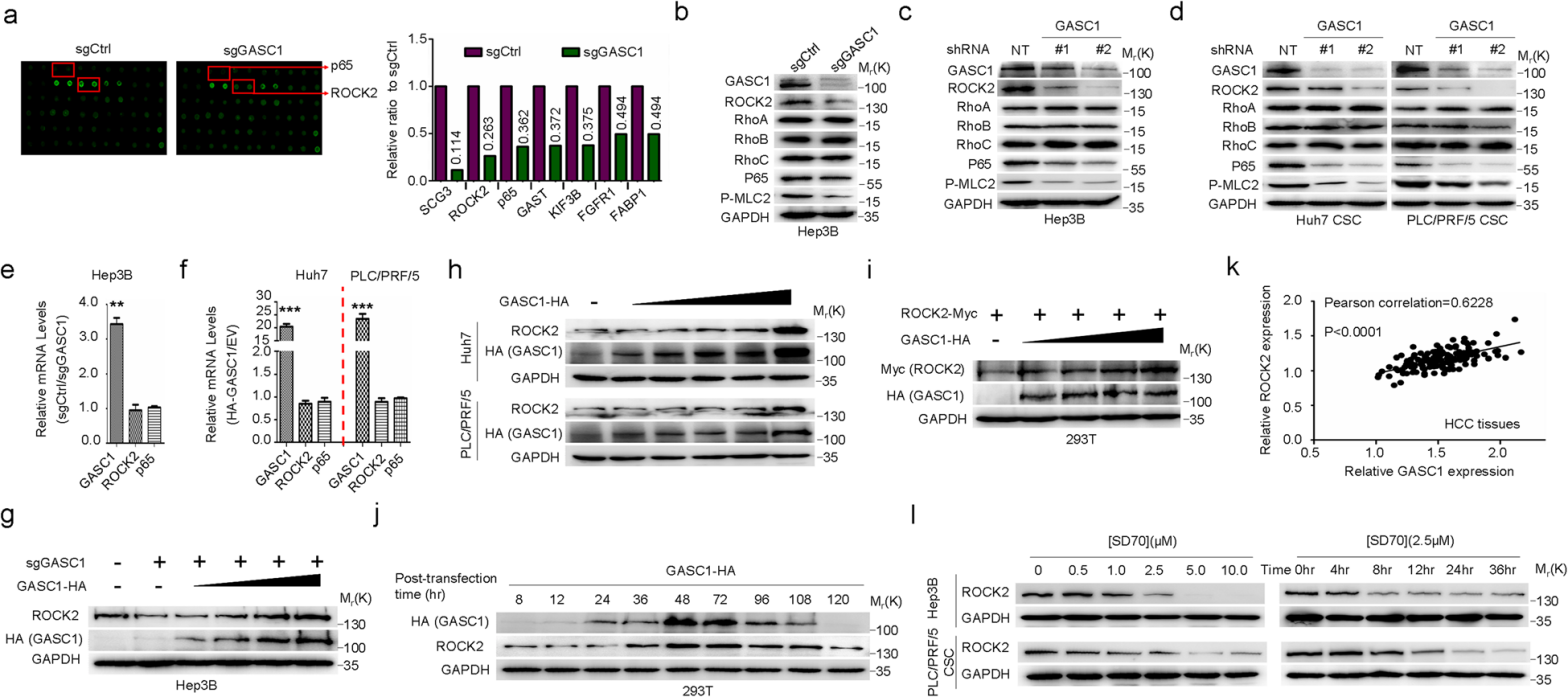
GASC1 positively regulates ROCK2 protein level through a mechanism dependent on its histone demethylase activity. **a** High-density protein microarray screening analysis of cell lysates from cultured control and GASC1-depleted Hep3B cells. Proteins significantly downregulated by *GASC1* depletion were summarized in the bar graph. **b** Immunoblotting (IB) analysis using the indicated antibodies in Hep3B GASC1 knockout (KO) cells. **c**, **d** IB analysis using the indicated antibodies in Hep3B (**c**), Huh7 CSC (**d**, left), and PLC/PRF/5 (**d**, right) CSC cells infected with shGASC1 and NT shRNA for 72 h. **e**, **f** qRT-PCR analysis of GASC1, ROCK2, and p65 mRNA levels in Hep3B GASC1 knockout cells (**e**) or HA-GASC1 over-expressed Huh7 and PLC/PRF/5 cells (**f**). Error bars represent SEM of three independent experiments. **P < 0.01 and ***P < 0.001 by Student’s *t* test. **g–i** IB analysis of whole-cell lysates (WCL) derived from Hep3B GASC1-KO cells (**g**), Huh7 and PLC/PRF/5 cells (**h**), and HEK293T cells (**i**) transfected with indicated plasmids for 48 h. **j** ROCK2 levels in HEK293T cells were monitored during the indicated time frame after transfection of HA-GASC1 plasmid (8 h to 120 h post transfection). **k** GASC1 and ROCK2 gene expression positively correlate with each other in HCC clinical specimens. **l** IB analysis of ROCK2 in Hep3B and PLC/PRF/5 CSCs after treatment with increasing doses of SD70, an inhibitor of the GASC1 histone demethylase activity, for 48 h (left panel); or 2.5 μM SD70 for increasing lengths of time as indicated (right panel).

Previous studies had shown that the enzymes modulating lysine methylation could regulate target protein levels without affecting their mRNA transcription^37,38^. To explore such mechanism for GASC1 in promoting HCC development, we introduced GASC1 KO into the GASC1^High^ cell line-Hep3B and conducted a high-density protein microarray screening of a panel of proteins implicated in tumor-stromal pathways. Among the top seven factors with the most reduced protein levels in GASC1 KO cells (Fig. 3a), only ROCK2 and p65 were previously implicated in HCC pathological development^7,39,40^. Therefore, we focused on the signaling pathway involving these two factors in our subsequent investigations. Using immunoblotting, we further confirmed the decreases in ROCK2, p65 protein levels, and the phosphorylation of myosin light chain 2 (P-MLC2, a specific downstream effector of ROCK2) (Fig. 3b, c) in Hep3B cells depleted of GASC1 by either CRISPR/Cas9 or shRNA. Given that ROCK2 is downstream of Rho family of GTPases (RhoA, RhoB, and RhoC) in the pathway, we checked the protein levels of the three members and the Rho-GTPase activity, our results showed no significant changes in these parameters upon GASC1 depletion by CRISPR/Cas9 (Fig. 3b, c, Supplementary Fig. 3). Similar results were obtained in the liver CSCs in which GASC1 is highly expressed (Fig. 3d). These results indicated that GASC1 regulates ROCK2 expression through a mechanism independent of the Rho GTPases. Intriguingly, we observed no obvious suppression of ROCK2 and p65 mRNA expression when GASC1 was depleted (Fig. 3e). Moreover, we also found that the ectopic expression of GASC1 had no effect on ROCK2 and p65 mRNA transcription (Fig. 3f). From a clinical perspective, we found no significant co-occurrence of GASC1 and ROCK2 at mRNA levels in TCGA liver cancer samples (442 patients) from the cbioportal database (http://www.cbioportal.org)^41^ (Supplementary Fig. 5f). These observations suggested that GASC1-medicated upregulation of ROCK2 and p65 protein levels are not the result of transcriptional regulation.

ROCK2 serves as a key regulator in proliferation, apoptosis, inflammatory responses, and metabolism. It is also a critical inducer of tumorigenesis^8,42,43^. In HCC, ROCK2 is highly expressed, which predicts poor outcomes in these patients^7,40^. Moreover, ROCK2 overexpression induces resistance to apoptosis and chemotherapy, thereby conferring tumor recurrence^6^. Aberrantly activated ROCK2 promotes NF-κB p65 subunit activation, which drives lysophosphatidic acid-mediated expression of cell adhesion factors^44^. Therefore, we examined the regulation of the ROCK2 pathway by GASC1.

To investigate the regulatory mechanism by GASC1 on ROCK2 protein expression, we noticed that the ectopic expression of GASC1 increased the abundance of ROCK2 protein levels in a dose-dependent manner (Fig. 3g, h). Similarly, we detected a marked increase of ROCK2 expression by co-transfecting HEK293T cells with GASC1 (Fig. 3i). We also monitored ROCK2 levels in HEK293T cells after transfection with HA-tagged GASC1 expression plasmid (Fig. 3j). We found that ROCK2 protein expression increased 24 h post transfection, coinciding with GASC1-HA induction. After 96 h, when GASC1-HA expression started to decline, ROCK2 levels decreased in parallel and, concomitant with the disappearance of GASC1-HA at 120 h, ROCK2 level also reached baseline. Subsequently, we investigated a potential link between GASC1 and ROCK2 protein levels in tumor specimens from HCC patients and identified a positive correlation between GASC1 and ROCK2 protein expression (Fig. 3k).

Recent studies have described small molecules that specifically inhibit GASC1 demethylase activity in vitro and in vivo^18^. Similar to the observation made in GASC1 knockdown cells, GASC1 inhibitor SD70 treatment decreased ROCK2 protein expression in a dose- and time-dependent manner (Fig. 3l). These data demonstrated that the demethylase activity of GASC1 is required for its function in promoting ROCK2 protein expression.

### GASC1 disrupts ROCK2 K63-linked poly-ubiquitylation to enhance ROCK2 protein stability

**Fig. 4:**
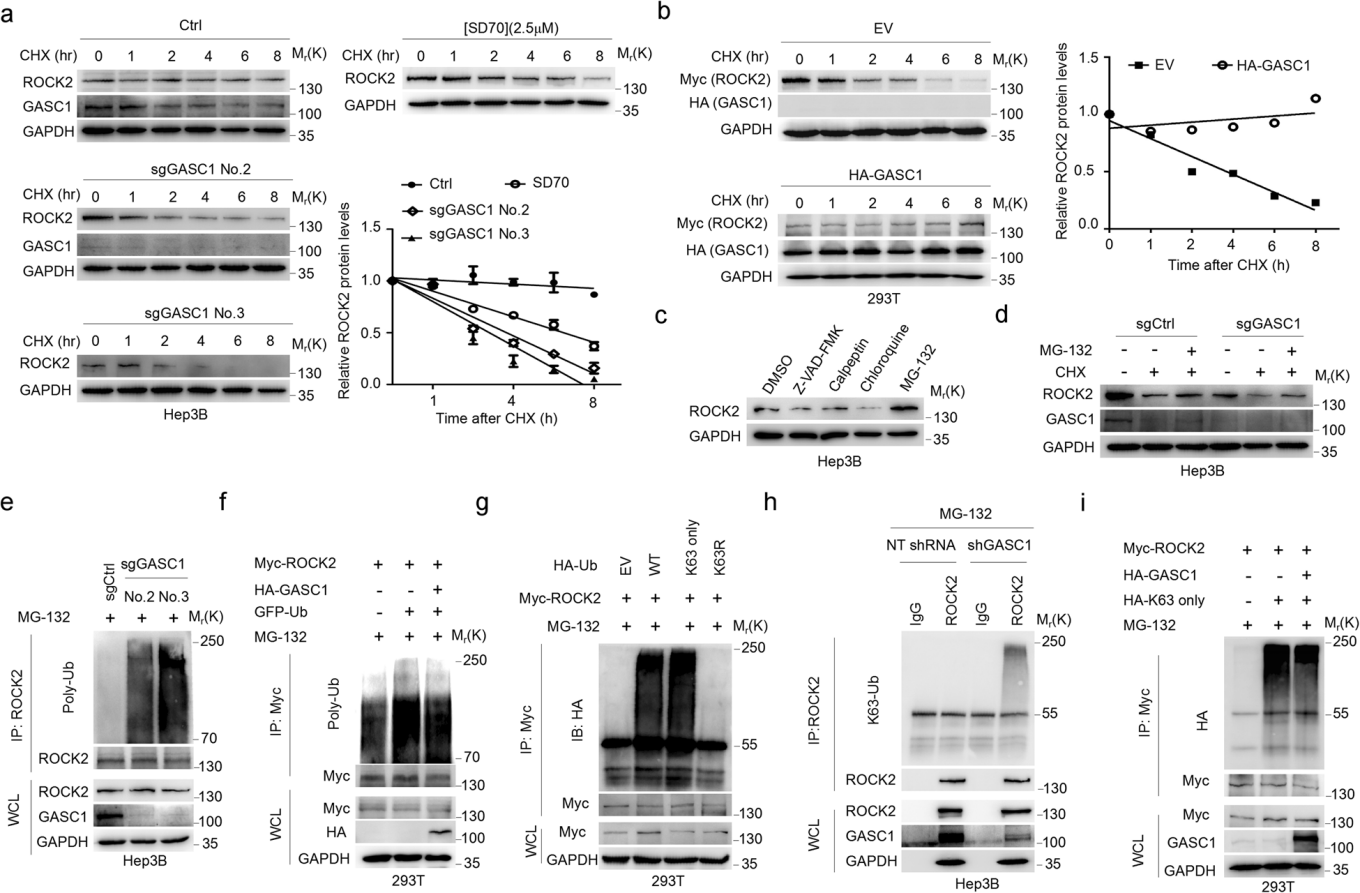
GASC1 stabilizes ROCK2 through preventing K63-linked poly-ubiquitination. **a** Immunoblotting (IB) analysis of ROCK2 stability in Hep3B GASC1-KO clones or WT cells treated with SD70 for 36 h. Where indicated, 100 μg/ml cycloheximide (CHX) was added, and cells were harvested for IB analysis at the indicated time points. The ROCK2 protein abundance was quantified by Image J and plotted in the graph. **b** IB analysis of ROCK2 stability in HEK293T cells transfected with the indicated plasmids. Cells were treated with 100 μg/ml CHX for the indicated time period before harvesting (left panel). The Myc-tagged (ROCK2) protein abundance was quantified by Image J and plotted as in the graph. EV empty vector. **c** IB analysis of ROCK2 protein levels in PLC/PRF/5 cells after treatment with 10 μM chloroquine, calpeptin, Z-VAD-FMK, or MG-132 for 24 h before harvesting. **d** IB analysis of ROCK2 protein levels in Hep3B cells with GASC1 depletion. Cells were treated with or without 100 μg/ml CHX and 10 μM MG-132 for 8 h before harvesting. **e** IB analysis of ROCK2 poly-ubiquitination in GASC1 knockout Hep3B cells. Cells were treated with 10 μM MG-132 for 6 h before harvesting. **f** IB analysis of ROCK2 poly-ubiquitination upon HA-GASC1 overexpression in HEK293T cells. Cells were treated with 10 μM MG-132 for 6 h before harvesting. **g** Significant levels of ROCK2 poly-ubiquitination can be detected by IB in cells transfected with wide-type ubiquitin or lysine-63-linked ubiquitin (K63 only), but not with lysine-63-arginine mutant ubiquitin (K63R) constructs. Cells were treated with 10 μM MG-132 for 6 h before harvesting. **h** *GASC1* knockdown increases K63-linked poly-ubiquitination of ROCK2 in Hep3B cells. Cells were treated with 10 μM MG-132 for 6 h before harvesting. **i** Poly-ubiquitination of ROCK2 inversely correlated with GASC1 ectopic expression. IB analysis of WCL and anti-Myc IP products derived from HEK293T cells transfected with HA-GASC1, Myc-ROCK2, and HA-Ub constructs. Cells were treated with 10 μM MG-132 for 6 h before harvesting.

We further investigated the molecular mechanism underlying the GASC1-mediated regulation of ROCK2 protein stability. Toward this end, cells were treated with cycloheximide (CHX) to block protein synthesis, then we examined the effects of GASC1 depletion/inhibition on ROCK2 protein stability. As shown in Fig. 4a, GASC1 depletion by CRISPR/Cas9 or inhibition by SD70 treatment substantially shortened the half-life of ROCK2 in relative to that in control cells. In contrast, ectopically expressed GASC1 markedly extended the half-life of ROCK2 in HEK293T (Fig. 4b). Next, we treated cells with inhibitors of the four major proteolytic pathways: proteasome (MG-132), lysosome (chloroquine), calpain (calpeptin), and caspase (Z-VAD-FMK). We found that MG-132 treatment induced the most significant accumulation of ROCK2 protein, whereas no significant effect was observed upon treatment with the other inhibitors (Fig. 4c, Supplementary Fig. 4a). Importantly, MG-132 treatment partially restored ROCK2 protein levels in GASC1-KO and SD70-treated cells (Fig. 4d, Supplementary Fig. 4b), indicating GASC1 KO and inhibition caused ROCK2 degradation through proteasome pathway. Moreover, we found that GASC1 inhibits intracellular ROCK2 protein poly-ubiquitination (Fig. 4e, f).

By analyzing the poly-ubiquitin chain type of ROCK2, we found that the K63-linked ubiquitin is the predominant form among the seven chain types examined (Supplementary Fig. 4c). Moreover, we further validated the result using a K63R mutant type of ubiquitin, which completely abolished ROCK2 poly-ubiquitination (Fig. 4g). In the loss- and gain-of-function studies of GASC1, we found that the levels of ROCK2 poly-ubiquitination anti-correlated with GASC1 protein levels in GASC1-depleted or over-expressed cells (Fig. 4h, i). The results indicated a novel role for K63-linked poly-ubiquitination of ROCK2 in modulating its protein stability.

### Deficiency in ROCK2 poly-ubiquitination caused by GASC1-depletion confers oncogenicity in HCC cells

**Fig. 5:**
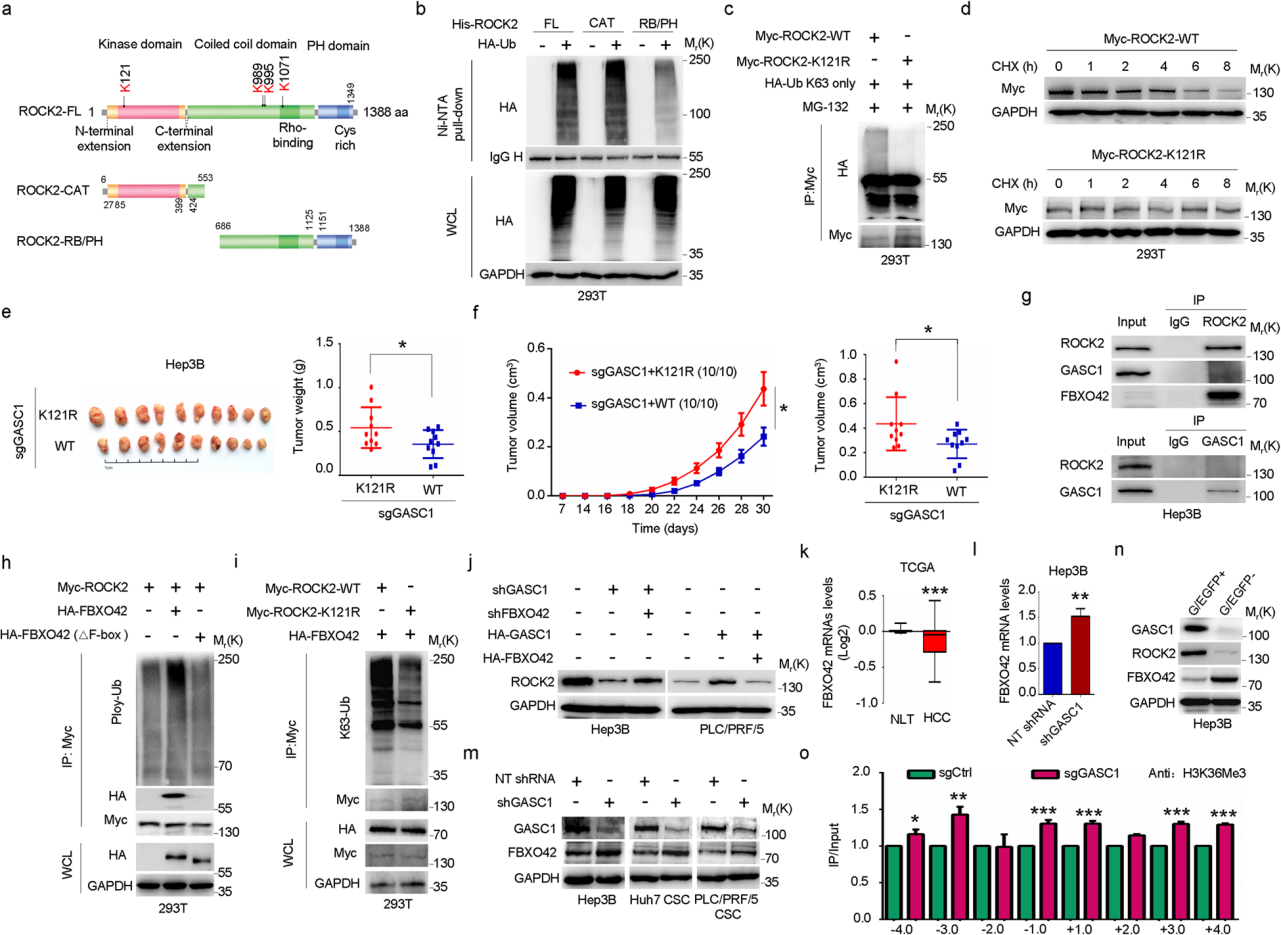
GASC1 epigenetically represses FBXO42 expression to prevent ROCK2 K121 poly-ubiquitination and degradation. **a** Schematic of ROCK2 functional domains with potential poly-ubiquitination sites indicated. **b** CAT domain harbors the main poly-ubiquitination sites in ROCK2. Cells were treated with 10 μM MG-132 for 8 h before harvesting. FL full length. CAT catalytic domain region. RB/PH Rho-binding/PH region auto-inhibitory domains. **c** Point mutation of K121 within the CAT domain abolishes ROCK2 poly-ubiquitination. Poly-ubiquitination of ROCK2 in HEK293T cells was examined by transfecting WT and K121R Myc-ROCK2 constructs together with HA-K63-linked ubiquitin, followed by IP and IB analyses. Cells were treated with 10 μM MG-132 for 6 h before harvesting. **d** K121R mutation significantly extended the half-life of ROCK2 protein. Cells were treated with 100 μg/ml CHX for the indicated time periods before harvesting. **e**, **f** Xenograft tumor growth of subcutaneous tumors formed by GASC1-depleted Hep3B cells stably expressing ROCK2 or ROCK2-K121R. Tumor image and tumor weight (**e**) were presented. Tumor growth was measured at the indicated time points and dissected at the endpoint (*n* = 10 tumors) (**f**). Data are represented as mean ± SEM. *P < 0.05 by two-way ANOVA. **g** ROCK2 or GASC1 was not detected in GASC1 or ROCK2 IP sample, respectively. FBXO42 was detected in ROCK2 IP sample by immunoblotting. Rabbit immunoglobulin G (IgG) was used as a negative control for the IP. **h** FBXO42 poly-ubiquitinates ROCK2 in vivo and the activity is dependent on its F-box domain. Cells were treated with 10 μM MG-132 for 8 h before harvesting. **i** K121R mutation largely abolishes FBXO42-mediated ROCK2 poly-ubiquitination. Cells were treated with 10 μM MG-132 for 8 h before harvesting. **j** IB analysis of ROCK2 protein levels in Hep3B and PLC/PRF/5 cells transfected with the indicated plasmids for 48 h. **k** Box plots indicate FBXO42 expression in normal liver tissues (NLT) and liver cancer tissues (HCC) from TCGA datasets. Comparison was performed using two-tailed Student’s *t* test. ***P < 0.001. **l** qRT-PCR analysis of FBXO42 mRNA level in Hep3B cells infected with NT shRNA or GASC1 lentiviral shRNA (shGASC1) vectors for 72 h before harvesting. Error bars represent SEM of three independent experiments. **P < 0.01 by Student’s *t* test. **m** IB analysis of FBXO42 protein levels in Hep3B, Huh7 and PLC/PRF/5 CSC cells upon GASC1 shRNA knockdown. The infected cells were selected with 1 μg/mL puromycin for 72 h to eliminate the non-infected cells before harvesting. **n** IB analysis of GASC1, ROCK2 and FBXO42 protein levels in FACS-sorted G/EGFP+ and G/EGFP− Hep3B subpopulations. **o** ChIP assay was performed to analyze the enrichment of H3K36Me3 at the transcriptional regulatory regions of the FBXO42 gene in Hep3B cells. Data are presented as mean ± SEM of three independent experiments. *P < 0.05; **P < 0.01; ***P < 0.0001 by Student’s *t* test.

ROCK2 is a member of the AGC kinase family, containing an N-terminal catalytic domain, a central coiled-coil domain, and a C-terminal PH domain carrying a Cys-rich region. The catalytic domain is responsible for Rho-kinase activity (ROCK2-CAT), which is blocked by intramolecular autoinhibition via the Rho-binding/PH region (ROCK2-RB/PH)^45^ (Fig. 5a). We found that the catalytic domain (amino acids 6-583) was both necessary and sufficient for ROCK2 ubiquitination in cells (Fig. 5a, b), despite that ROCK2-RB/PH contains three potential ubiquitination sites (K989, K995, and K1071; http://www.phosphosite.org)^46^. Next, we sought to identify the critical ROCK2 ubiquitination site within the catalytic domain. We found that mutating the conserved lysine residue K121 (Supplementary Fig. 5a) strongly reduced cellular levels of K63-linked ubiquitination on ROCK2 (Fig. 5c). We further observed that the ubiquitination-deficient K121R mutant protein (ROCK2-K121R) had a significantly longer half-life as compared with that of the wild-type (WT) protein (Fig. 5d, Supplementary Fig. 5b).

Strikingly, in GASC1-depleted cells, the expression of ubiquitination-deficient ROCK2-K121R mutant significantly enhanced tumor growth, as compared to that of WT (Fig. 5e, f). These results suggested that the K121R mutation stabilizes ROCK2 and stimulates its oncogenic activity, in part by slowing down its enhanced turnover under cellular GASC1 deficiency.

### GASC1 inhibits poly-ubiquitination of ROCK2 via transcriptional repression of FBXO42

To understand how GASC1, a histone demethylase controls ROCK2 stability through poly-ubiquitination, we first asked whether there exists a direct GASC1-ROCK2 interaction. However, we did not detect ROCK2-GASC1 endogenous interaction in Hep3B (Fig. 5g) or exogenous interaction in HEK293T cells (Supplementary Fig. 5c) using reciprocal co-immunoprecipitation assay. To identify the GASC1 modulated ubiquitin ligase(s) that regulates ROCK2 turnover, we screened through the potential interaction partners of ROCK2, which had been reported in BioGRID (https://thebiogrid.org/). Among the 27 previously identified ROCK2-binding partners, only FBXO42 is a protein-ubiquitin ligase. Previous studies have demonstrated that FBXO42 regulates p53/TP53 or ING4 protein abundance via the ubiquitin–proteasome pathway, and the activity depends on its F-box domain^47,48^. Using an immunoprecipitation assay, we confirmed that ROCK2 binds to FBXO42 in vivo (Fig. 5g), leading us to hypothesize that increased FBXO42 in response to GASC1 deficiency could promote ROCK2 protein degradation. Interestingly, we verified that FBXO42 promoted ROCK2 protein degradation in a dose-dependent manner, which could be efficiently blocked by MG-132 (Supplementary Fig. 5d, e). Moreover, we found that the deletion of the F-box domain in FBXO42 abolished its interaction with ROCK2 and subsequent ROCK2 ubiquitination and degradation (Fig. 5h). In accordance with these findings, unlike the WT protein, the ROCK2-K121R mutant was resistant to FBXO42-mediated K63-linked ubiquitination (Fig. 5i). Similarly, the effects of GASC1 knockdown or overexpression on ROCK2 protein levels were abolished by silencing or ectopic expression of FBXO42, suggesting that FBXO42 is a critical downstream effector of GASC1 in the pathway of ROCK2 regulation (Fig. 5j). In summary, these results indicated that the K121 residue is the major ubiquitination site for FBXO42-mediated ROCK2 ubiquitination and degradation in GASC1-deficient cells.

As a poor-prognosis marker, high ROCK2 level was reported to correlate with adverse outcomes in HCC patients^7,40^. Therefore, we hypothesized that a high FBXO42 level potentially promoting ROCK2 degradation could be a good-prognosis factor. Indeed, using the TCGA HCC data, we found that FBXO42 gene expression was highly elevated in normal liver tissues (NLT), compared with that in HCC (Fig. 5k).

Next, we sought to explore whether GASC1 loss-of-function correlates with elevated FBXO42 expression in HCC by analyzing TCGA liver cancer samples (442 patients) from the cBio database. We found that GASC1 and FBXO42 mRNA expression are mutually exclusive (Supplementary Fig. 4f), suggesting that GASC1 may contribute to the decreased FBXO42 transcription. We tested this hypothesis and found that GASC1 negatively regulated FBXO42 mRNA and protein expression (Fig. 5l, m, Supplementary Fig. 5g, h). In addition, we also observed that FBXO42 was highly expressed in G/EGFP− populations, but not in G/EGFP+ populations (Fig. 5n). To gain further mechanistic insights, we performed ChIP assay to determine whether GASC1 regulates FBXO42 transcription through histone demethylation. It has been demonstrated that GASC1 functions as the demethylase for the trimethylation mark on histone H3 lysine 9 (H3K9me3) and lysine 36 (H3K36me3)^15,49,50^. Our results showed that in GASC1-deficient cells, the FBXO42 promoter region was highly enriched in H3K36me3 (Fig. 5o). These results indicated that GASC1 transcriptionally represses FBXO42 expression by erasing the active chromatin mark of H3K36me3 from its promoter, which subsequently prevents ROCK2 poly-ubiquitination and degradation.

### SD70 inhibits HCC growth in vitro and in vivo and enhances chemo-sensitivity

**Fig. 6:**
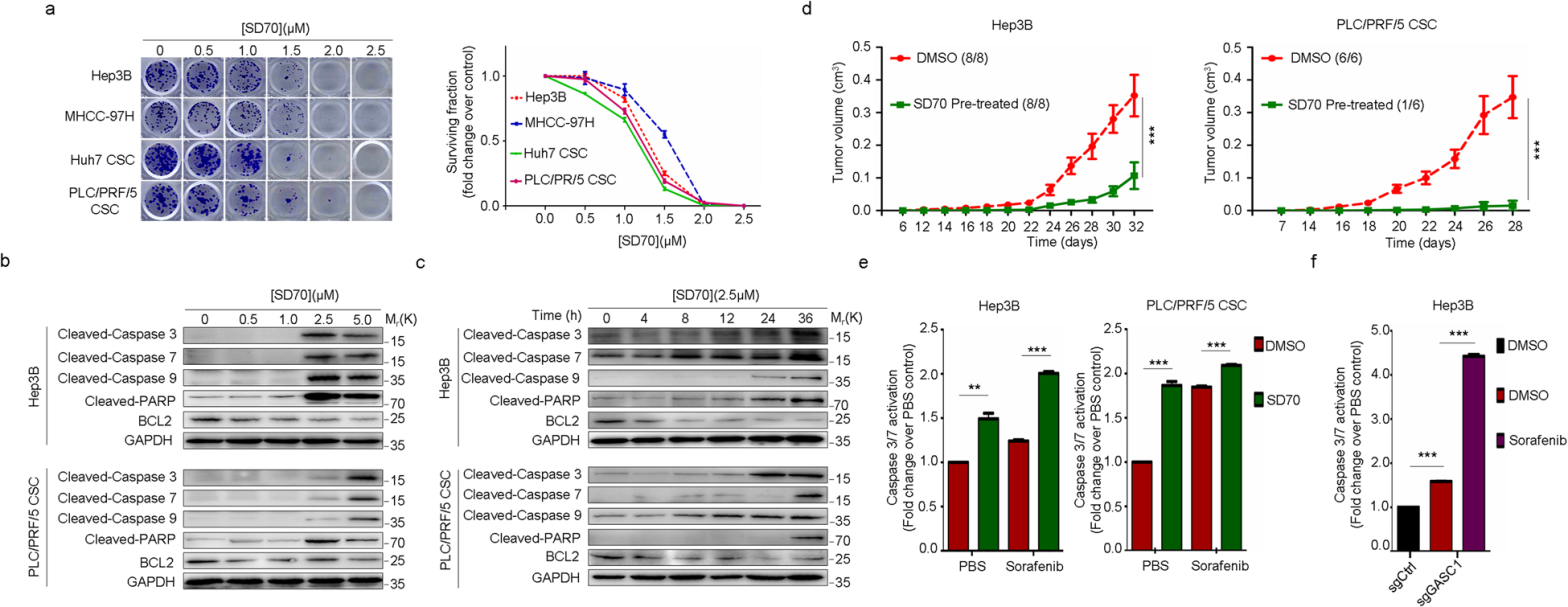
GASC1 inhibitor SD70 impairs clonogenic survival of liver cancer cells and tumor growth in vivo in xenograft models of HCC. **a** Clonogenic survival and growth of Hep3B, MHCC-97H, Huh7 CSC and PLC/PRF/5 CSC cells after treatment with increasing concentrations of SD70, as assessed by colony-formation assays (left panel), and quantification of the surviving fraction values for each condition, determined from the number of colonies for each condition relative to those in the blank control (right panel). Data are presented as mean ± SEM of three independent experiments. **b**, **c** Immunoblotting analysis of the indicated protein levels in Hep3B and PLC/PRF/5 CSC cells treated with increasing concentrations of SD70 (**b**), or 2.5 μM SD70 for increasing amount of time (**c**). **d** Xenograft tumor growth of subcutaneously implanted Hep3B and PLC/PRF/5 CSC cells pre-treated with SD70 or vehicle control. Tumor growth was monitored at the indicated time points and tumors were dissected at the endpoint. Data are presented as mean ± SEM. The number of treated mice (*n*) per group was shown. Data from the last time point was analyzed by two-tailed unpaired *t* test. (***P < 0.001). **e**, **f** Caspase-3/7 activation levels in Hep3B and PLC/PRF/5 CSC cells following 48 h of incubation with sorafenib, in the presence or absence of 2.5 μM SD70 (**e**) or under the GASC1 knockout condition (**f**). Data are presented as mean ± SEM from three independent experiments. Data were analyzed by two-tailed unpaired *t* test, **P < 0.01; ***P < 0.001.

**Fig. 7:**
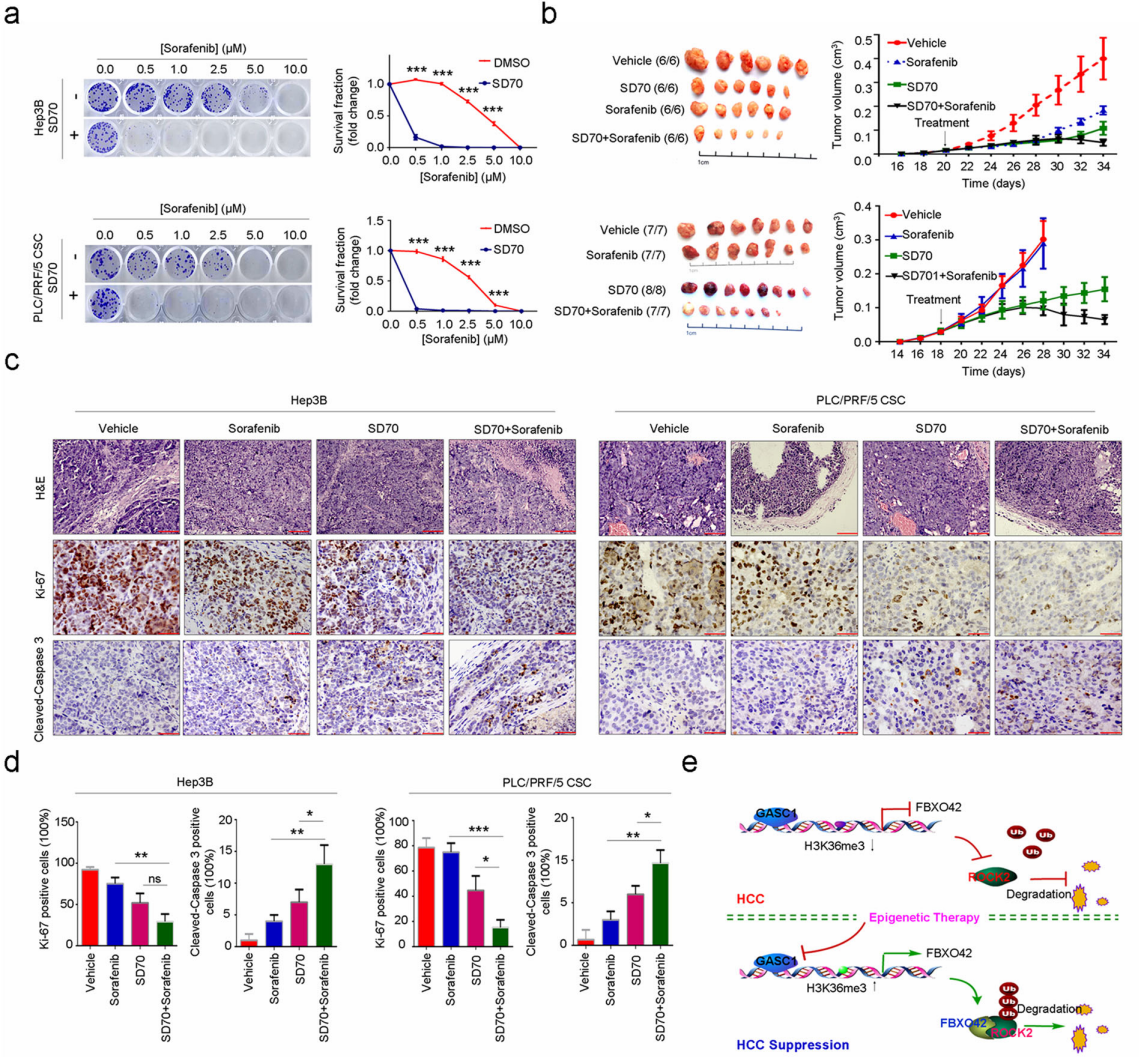
The specific GASC1 inhibitor SD70 enhances responses to chemotherapy in HCC cells and xenografts. **a** Representative images of colony-formation assays (left panel) and quantification of surviving fraction values (relative to the number of colonies in the DMSO control group) (right panel) for the indicated cell lines after treatment with increasing doses of sorafenib in the presence or absence of 2.5 μM SD70. Data are presented as mean ± SEM. ***P < 0.01 was determined by the paired *t* test. **b** Tumor volume over time in mice bearing Hep3B (upper panel) or PLC/PRF/5 CSC (lower panel) xenografts following treatment with sorafenib by oral gavage (OG), SD70 by intraperitoneal injection (IV), or a combination of sorafenib and SD70. Data are presented as mean ± SEM. *P < 0.05; **P < 0.01; ***P < 0.001 were determined by two-way ANOVA. **c** Representative H&E- and IHC-staining images of Hep3B and PLC/PRF/5 CSC xenografts (vehicle-treated, sorafenib treated, SD70-treated, and SD70 + sorafenib treated) at the end of the indicated treatments. Scale bars, 50 μm (H&E-staining) or 100 μm (Ki-67 and Cleaved-Caspase-3 IHC-staining). **d** Quantification of cell density (number of tumor cells/area) of Hep3B and PLC/PRF/5 CSC xenografts at the end of the indicated treatments. Each symbol represents an individual tumor. Data were analyzed by one-way ANOVA with Tukey’s multiple-comparisons test. *P < 0.05; **P < 0.01; ***P < 0.001. **e** A working model illustrating the roles of GASC1/FBXO42 axis-controlled ROCK2 protein poly-ubiquitination and degradation, which suppresses HCC development.

If GASC1 activity was essential for HCC progression, targeting this enzyme would represent an attractive therapeutic strategy. To evaluate the potential of SD70 in targeting GASC1-dependent HCC growth, we tested the capability of HCC cell lines in colony-formation under SD70 treatment. Our results indicated that SD70 treatment significantly decreased the capacity of colony-formation of GASC1^High^ and CSC cells colony-formation capacity in a dose-dependent manner (Fig. 6a). SD70 was shown to induce apoptosis in acute myeloid leukemia^14^. Consistent with this report, SD70 treatment strongly induced apoptosis as evidenced by the increased apoptotic markers Cleaved-Caspase-3, 7, 9, Cleaved-PARP and decreased antiapoptotic gene BCL2 expression in both dose- and time-dependent manners (Fig. 6b, c). We also tested the effect of SD70 treatment on mice bearing xenografts derived from GASC1^High^ cells and CSC populations. We found SD70 treatment substantially impaired tumor development in vivo (Figs. 6d, 7b).

Induction of apoptosis is critical for the success of chemotherapy, which remains the mainstay of HCC treatment despite limited efficacy^51^. Treatment of HCC cells with the standard advanced HCC chemotherapy agent sorafenib increased caspase-3/7 activity. This effect was further enhanced by SD70 treatment (Fig. 6e) or GASC1 knockout (Fig. 6f) in Hep3B and PLC/PRF/5 CSC populations. We then investigated the therapeutic potential of combining GASC1 inhibition with a chemotherapeutic agent in HCC. Interestingly, SD70 treatment sensitized the cells to sorafenib in the colony-formation assay (Fig. 7a). The efficacy of combination therapy in reducing long-term colony formation in GASC1-sensitive cells was consistent with the observation that the addition of SD70 to standard-of-care chemotherapy increased caspase-3/7 activity (Fig. 6e). In summary, these findings suggested that the effect of the combination therapy on colony formation was mediated by the induction of apoptosis.

We then tested the potential of GASC1 inhibition to sensitize HCC xenografts in mice to chemotherapy. Xenografts generated from two cell lines were treated with sorafenib and SD70. SD70 treatment alone led to disease stabilization, whereas the combination of sorafenib and SD70 led to increased treatment responses in the xenografts (Fig. 7b) and a marked reduction in viable tumor cell proliferation, along with an increase in apoptosis at the tumor implantation site (Fig. 7c, d), demonstrating the potential efficacy of combining GASC1 inhibition with sorafenib treatment.

## Discussion

Elucidation of the mechanisms that coordinate epigenetic reprogramming by oncogenic chromatin modifiers is critical for our understanding of the molecular biology of cancer and the development of effective therapeutic strategies^52^. Our results identified GASC1 as an apical regulator of HCC progression that confers proliferative advantage to the tumor by protecting ROCK2 protein stability, which further leads to shortened survival time in HCC patients. Furthermore, our mechanistic and clinical findings established that GASC1 controls ROCK2 ubiquitination and degradation through epigenetic repression of its critical ubiquitin ligase FBXO42 (Fig. 7e). Through complementary approaches of GASC1 depletion by shRNA and inhibition by SD70, we demonstrated that GASC1 activity is required for the progression of HCC both in culture and in vivo, especially under the condition of chemotherapy, indicating that GASC1 is a potential therapeutic target in HCC.

Accumulation of genetic mutations and epigenetic changes in regenerating mature hepatocytes during a chronic liver injury leads to HCC development^53^. However, growing evidence favors the hypothesis that CSCs (or TICs) represent a rare subpopulation within the tumor and are responsible for tumor initiation and chemoresistance^54,55^. Using in vitro cell line models, GASC1 has been implicated in different cancers including B-cell lymphoma^10^, acute myeloid leukemia^14^, and prostate^15^ cancers. In this report, we show that high GASC1 levels in HCC are associated with increased tumor progression. Further examination discovered that GASC1 was essential for cell growth and tumor growth in GASC1^High^ cells, including CSC-like cells, but was expendable in GASC1^Low^ cells, including non-CSCs-like cells. Indeed, although the upregulation of GASC1 expression mostly driven by copy-number aberration was specifically associated with CSC-like cells, which form a small subset of HCC, the function of GASC1 may be relevant to a broader range of initiating HCC cells.

It has been reported that ROCK2 is cleaved by granzyme B during granule-mediated killing of target cells^8^. In this study, we found that cellular ROCK2 level is enhanced by GASC1 in an indirect, transcription-dependent manner. The involvement of GASC1 in regulating the stability of ROCK2 is intriguing. The posttranslational regulation of ROCK2 mediated by GASC1/FBXO42 axis provides a delicate control of ROCK2 protein stability during HCC initiation and tumor growth. The regulation of protein turnover via the ubiquitin–proteasome system affects diverse aspects of eukaryotic biology. Gain- or loss-of-function mutations of specific components of the Skp1/Cullin/F-box (SCF)-E3 ubiquitin ligase complex has direct implications for tumorigenesis^56^. Substrate specificity is conferred by the E3 ubiquitin ligases, of which the SCF family with Cullin-RING ubiquitin ligases (CRLs) is among the most intensively studied^57,58^. So far, only a few substrates of FBXO42 have been identified besides ROCK2^47,59^, and little is known about the function of FBXO42 in HCC. Our study demonstrated that the expression of FBXO42 is low in HCC and that its main function appears to be the regulation of ROCK2 abundance by mediating ubiquitination and degradation. In earlier reports, it has been shown that the loss of SETD2 resulted in a reduction of the H3K36me3 mark and accelerated tumor development^60,61^, suggesting a tumor-suppressor activity associated with H3K36me3 functioning in a critical but unknown locus. Consistent with this hypothesis, we found that recruitment of GASC1 by chimeric transcription factors counteracted and removed the active H3K36 trimethylation mark associated with target gene loci such as FBXO42, which is critical for ROCK2 ubiquitination and degradation. However, whether GASC1 regulates other ubiquitinases via a similar mechanism requires a systematic and thorough investigation.

Importantly, our findings provided strong in vitro and preclinical in vivo evidence for the efficacy of HCC suppression by pharmacological inhibition of GASC1 via a small molecule inhibitor. Thus, we generated critical data for future clinical testing of GASC1 inhibitors in human cancer treatment. Interestingly, GASC1-null mice developed normally and are fertile^17^, indicating that GASC1 may not be essential for physiologic tissue homeostasis, which could significantly lower burdens of GASC1 inhibition and extend the disease latency.

In summary, our study provided insights into the mechanisms employed by GASC1 to propagate malignant phenotypes in HCC and provides a rationale for clinical trials to investigate GASC1 inhibition combined with chemotherapy as a novel therapeutic strategy in HCC with high levels of *GASC1* gene expression. Moreover, GASC1 inhibition may provide a much-needed treatment option for tumors that stably express ROCK2, as well as other antiapoptotic BCL2 family members that mediate resistance to liver cancer chemotherapy.

## Supplementary information

Supplementary Information
