# Supplementary material for: GASC1 promotes hepatocellular carcinoma progression by inhibiting the degradation of ROCK2
Source: Cell Death Dis. 2021 Mar 10;12(3):253. doi: 10.1038/s41419-021-03550-w (PMC7946911; doi:10.1038/s41419-021-03550-w)
Supplement: Supplementary file 1 — Supplementary Information [file 41419_2021_3550_MOESM1_ESM.doc]

**Supplementary Information**

**Supplementary Table S1. A list of primers used for RT-PCR in this study.**

| Gene | Forward | Reverse |
| --- | --- | --- |
| ROCK2 | 5’-TGGTTTCTATGGGCGAGAATGT-3’ | 5’-CAAGTCGTACCTCCCTATCTGTT-3’ |
| P65 | 5’-ATGTGGAGATCATTGAGCAGC-3’ | 5’-CCTGGTCCTGTGTAGCCATT-3’ |
| FBXO42 | 5’-TCTGCAAACAGTGGTATCGAC-3’ | 5’-TTCCAGGATAAGGATAGGTACGG-3’ |
| GAPDH | 5’-TGGTATCGTGGAAGGACTC-3’ | 5’-AGTAGAGGCAGGGATGATG-3’ |

**Supplementary Table S2. A list of primers used for ChIP in this study.**

| Gene | Forward | Reverse |
| --- | --- | --- |
| FBXO42 (-4.0) | 5’-GCCAAATCCAGCTTTTCCCA-3’ | 5’-AGCCAGCCTCCTTACAGATG-3’ |
| FBXO42 (-3.0) | 5’-GCCTGCCCATTCTTCTGTCT-3’ | 5’-AGGCTTGGTGATGCATGTG-3’ |
| FBXO42 (-2.0) | 5’-CATCCGGCCTTCTATCTTCTT-3’ | 5’-AATCATTAAGAGATCCAGGCAGT-3’ |
| FBXO42 (-1.0) | 5’-TGGGGATGGGCTTGGTATTT-3’ | 5’-GACATTCCACTCAACAGCTGT-3’ |
| FBXO42(+1.0) | 5’-CAGTTTCATGGCTGTGGACC-3’ | 5’-AGTCGATACCACTGTTTGCA-3’ |
| FBXO42 (+2.0) | 5’-GCAGTTAGTGGCTTCATTGC-3’ | 5’-CCATGAGAACCACCTTGATGC-3’ |
| FBXO42 (+3.0) | 5’-ACAAATTCCTCTGAGCGGAC-3’ | 5’-GTTTGAACGTTTCTCCTGCC-3’ |
| FBXO42 (+4.0) | 5’-CCTCCTCAGCCACCAGAATA-3’ | 5’-AAAGAAATGGAGGAAGCCGG-3’ |

**Supplementary Fig. 1.** GASC1 is essential for HCC proliferation and tumorigenesis.

**a** Correlation between copy number variation of GASC1 gene and its mRNA expression in liver cancer data from The Cancer Genome Atlas (TCGA).

**b** Histogram plots indicating *GASC1* mRNA expression in different HCC cell lines from the GSE36138 dataset.

**c** Immunoblotting (IB) analysis of GASC1 protein levels in various liver cancer cell lines.

**d** Growth curves for various liver cancer cells measured by cholecystokinin-8 (CCK-8) after infection with lentivirus expressing shGASC1. Data are presented as mean ± SEM of three independent experiments. **P*<0.05, and ***P*<0.01 by Student’s *t*-test.

**e** IB analysis of GASC1 protein levels in Hep3B cells with GASC1 knockout through CRISPR/Cas9 technology. Parental Hep3B cells were used as the control.

**f** IB analysis of GASC1 protein levels in PLC/PRF/5 cells stably over-expressing GASC1.

**g** Over-expression of GASC1 in PLC/PRF/5 cells increased tumor volume and tumor weight in NOD/SCID mice following xenotransplantation. Data are presented as mean ± SEM.*P<0.05, and ***P<0.0001 by Student’s t-test.

**Supplementary Fig. 2.** GASC1 is highly expressed in liver CSCs and is essential for the maintenance of liver CSCs self-renewal.

**a** Immunoblotting (IB) analysis of GASC1 expression in PLC/PRF/5 CSCs or non-CSCs cells sorted with indicated CSC markers (CD13, CD13, EpCAM, and CD24).

**b** IB analysis of GASC1expression in oncosphere and non-oncosphere cells derived from various HCC cell lines.

**c** IB analysis of GASC1 in liver CSC and non-CSC tumors derived from PLC/PRF/5 cells.

**d** Cell proliferation of PLC/PRF/5 CSC cells with GASC1 shRNA knockdown was measured by CCK-8 analysis. Data are presented as the mean ± SEM of three independent experiments. ***P*<0.01 by Student’s *t*-test.

**e** Immunofluorescence (IF) staining of Ki-67 in PLC/PRF/5 CSC cells with GASC1 shRNA knockdown. Scale bars, 50 μm.

**f, g** Clone formation efficiency (f) and sphere formation efficiency (g) assays in liver CSCs expressing shGASC1 for 2 weeks. Data are presented as mean ± SEM of three independent experiments. ***P*<0.01, and ****P*<0.0001 by Student’s *t*-test.

**h** GASC1 knockdown in PLC/PRF/5 CSC cells reduces xenograft tumor growth in NOD/SCID mice. Data are presented as mean ± SEM. N=6 for NT shRNA group and N=7 for shGASC1 group. ****P*<0.001 by non-parametric test.

**i** Limiting dilution assay of PLC/PRF/5 CSC cells with GASC1 shRNA knockdown in NOD/SCID mice (N=6/group). CSC frequency was determined by the software ELDA (http://bioinf.wehi.edu.au/software/elda).

Supplementary Fig. 3. GASC1 is not involved in Rho-GTPase activity in Hep3B cells.

Immunoblotting (IB) analysis of Rho-GTPase activity with GASC1 knockout in Hep3B cells through CRISPR/Cas9 technology.

**Supplementary Fig. 4.** GASC1 inhibits K63-linked poly-ubiquitination of ROCK2.

**a** Immunoblotting (IB) analysis ROCK2 protein levels in PLC/PRF/5 cells after treatment with 10 μM chloroquine, calpeptin, Z-VAD-FMK, or MG-132 for 24 h, then with 100 μg/ml cyloheximide (CHX) for 8 h before harvesting.

**b** IB analysis of ROCK2 protein levels in Hep3B cells treated with 2.5 μM SD70 for 36 h, then with indicated CHX and MG-132 treatment for 8 h before harvesting.

**c** ROCK2 poly-ubiquitination linkage was examined by transfecting HA-tagged wild-type and indicated lysine (6, 11, 27, 29, 33, 48, 63) only ubiquitin mutants together with Myc-ROCK2 into HEK293T cells, which was followed by IB analysis of anti-Myc IP products and whole-cell lysates. Cells were treated with 10 μM MG-132 for 6 h before harvesting.

**Supplementary Fig. 5.** GASC1 inhibits poly-ubiquitination and degradation of ROCK2 via transcriptional repression of FBXO42.

**a** A schematic diagram showing one evolutionarily conserved lysine residue (K121) within the catalytic domain of ROCK2. Hs: Homo Sapiens (human); Mm: Mus musculus (mouse); Cl: Canis lupus familiaris (dog); Pt: Pan troglodytes (chimpanzee); Rn: Rattus norvegicus (Norway rat); Bt: Bos taurus (bull).

**b** The Myc-tagged (ROCK2) protein abundance in Figure 5D was quantified by Image J and plotted as indicated.

**c** Co-IP analysis of Myc-ROCK2 and HA-GASC1 interaction in HEK293T cells.

**d** IB analysis of ROCK2 protein levels in HEK293T cells expressing increasing amounts of HA-FBXO42.

**e** HEK293T cells were transfected with indicated plasmids for 36 h and then treated with 20 μM MG-132 for 6 h. Cell lysates were immunoblotted with the indicated antibodies to evaluate the corresponding protein levels.

**f** Bioinformatics analysis of the cbioportal database (a database maintained by Memorial Sloan-Kettering) showed the frequency of GASC1, ROCK2, and FBXO42 mRNA expression, mutation, or amplification, or deletion events in HCC.

**g** qRT-PCR analysis of GASC1 and FBXO42 mRNA expression in HEK293T cells transfected with HA-GASC1 for 48 h. Data are presented as the mean ± SEM of three independent experiments. ****P* < 0.001 by Student’s *t*-test.

**h** IB analysis of FBXO42 protein levels in HEK293T and PLC/PRF/5 cells with HA-GASC1 over-expression.
